# TP53 /KRAS Co-Mutations Create Divergent Prognosis Signatures in Intrahepatic Cholangiocarcinoma

**DOI:** 10.3389/fgene.2022.844800

**Published:** 2022-03-25

**Authors:** Chunguang Guo, Zaoqu Liu, Yin Yu, Yunfang Chen, Hui Liu, Yaming Guo, Zhenyu Peng, Gaopo Cai, Zhaohui Hua, Xinwei Han, Zhen Li

**Affiliations:** ^1^ Department of Endovascular Surgery, The First Affiliated Hospital of Zhengzhou University, Zhengzhou, China; ^2^ Department of Interventional Radiology, The First Affiliated Hospital of Zhengzhou University, Zhengzhou, China; ^3^ Department of Pathophysiology, School of Basic Medical Sciences, The Academy of Medical Science, Zhengzhou University, Zhengzhou, China; ^4^ Department of Oncology, Zhumadian Central Hospital Affiliated to Huanghuai University, Zhumadian, China; ^5^ Department of Nursing, Zhumadian Central Hospital Affiliated to Huanghuai University, Zhumadian, China

**Keywords:** intrahepatic cholangiocarcinoma, mutation, TP53, KRAS, prognosis, TMB, chemotherapy

## Abstract

**Background:** Due to high invasiveness and heterogeneity, the morbidity and mortality of intrahepatic cholangiocarcinoma (ICC) remain unsatisfied. Recently, the exploration of genomic variants has decoded the underlying mechanisms of initiation and progression for multiple tumors, while has not been fully investigated in ICC.

**Methods:** We comprehensively analyzed 899 clinical and somatic mutation data of ICC patients from three large-scale cohorts. Based on the mutation landscape, we identified the common high-frequency mutation genes (FMGs). Subsequently, the clinical features, prognosis, tumor mutation burden (TMB), and pharmacological landscape from patients with different mutation carriers were further analyzed.

**Results:** We found TP53 and KRAS were the common FMGs in the three cohorts. Kaplan–Meier survival curves and univariate and multivariate analysis displayed that TP53 and KRAS mutations were associated with poor prognosis. Considering the co-mutation phenomenon of TP53 and KRAS, we stratified patients into “Double-WT,” “Single-Hit,” and “Double-Hit” phenotypes by mutation status. Patients with the three phenotypes showed significant differences in the mutation landscape. Additionally, compared with “Double-WT” and “Single-Hit” phenotypes, patients with “Double-Hit” presented a dismal prognosis and significantly high TMB. Through chemotherapy sensitivity analysis, we identified a total of 30 sensitive drugs for ICC patients, of which 22 were drugs sensitive to “Double-WT,” 7 were drugs sensitive to “Double-Hit,” and only one was a drug sensitive to “Single-Hit.”

**Conclusion:** Our study defined a novel mutation classification based on the common FMGs, which may contribute to the individualized treatment and management of ICC patients.

## Introduction

Intrahepatic cholangiocarcinoma (ICC), a primary malignant tumor derived from the bile ducts, has high invasiveness and heterogeneity (Moeini et al., 2016; Rizvi et al., 2018). In recent decades, ICC has attracted increasing global attention due to its difficult diagnosis, high morbidity, and poor prognosis features (Zou et al., 2021). Despite continued advances in the modalities of treatment, there is limited improvement in the overall survival (OS) of ICC patients (Moeini et al., 2016; Sirica et al., 2019; Kelley et al., 2020). The maximum OS of advanced ICC has not exceeded 15 months and the 5-year survival rate of ICC is under 10% (Antwi et al., 2018). The genetic heterogeneity of ICC is an important cause of its high malignancy (Sirica et al., 2019). Therefore, it is necessary to recognize “high-risk” patients based on genomic alterations of ICC, which will facilitate improve prognosis and personalized treatment.

With the development of high-throughput sequencing technologies and bioinformatics, the genomic characteristics of ICC were proved to correlate with prognosis (Lamarca et al., 2020). For example, the extracellular domain in-frame deletions of FGFR2 promoted the progression of cholangiocarcinoma and served as a genomic alteration of targeted therapy (Cleary et al., 2021). Zhou et al. reported that SLIT2 was identified as a driver of ICC dissemination and inflammatory cell infiltration (Zhou et al., 2021). Additionally, tumor mutation burden (TMB) as a novel mutational signature guides the prognosis of multiple solid tumors. Based on the International Cancer Genome Consortium (ICGC) database and the Memorial Sloan Kettering (MSK) Cancer Center, the comprehensive mutational characterization of ICC has been well described. Researchers have made numerous efforts to reveal tumor-associated drivers such as TP53, KRAS, ARID1A, IDH1, and SMAD4. Mutations of these drivers were involved in the progression, prognosis, immunotherapy, and targeted therapy (Liu et al., 2021a). Herein, we conjecture that some high-frequency mutation genes (FMGs) may play an important role in the prognosis of ICC. Compared with the existing prognosis signatures, FMGs do not require a defining cutoff value to stratify patients due to their binary data characteristics, which is more conducive to the cross-platform promotion and clinical application.

In this study, we identified FMGs in ICC patients based on multiple large-scale mutation cohorts. Then, based on the common FMGs (TP53 and KRAS) of three cohorts, we formulate three novel mutation phenotypes (“Double-WT,” “Single-Hit,” and “Double-Hit”), and the relationship of three mutation phenotypes with TMB and OS was further explored. Finally, we identified multiple chemotherapeutic drugs with specific sensitivity between the three phenotypes. Findings from our work may be conducive to the identification of “high-risk” ICC patients and the application of precise chemotherapy in clinical practice.

## Materials and Methods

### Data Collection and Processing

Somatic gene mutation data of three independent cohorts were collected from the cBioPortal dataset (https://www.cbioportal.org/), including the ICGC dataset, MSK-2021 dataset, and Shanghai dataset (Zou et al., 2014). The inclusion criteria for ICC cohorts and samples were as follows: 1) the sample size of the cohort was over 100; 2) selected the most recent cohort from the same institution for inclusion in the study; 3) have somatic mutation data; and 4) all were intrahepatic cholangiocarcinoma. A total of 899 patients (ICGC: 417; MSK-2021: 379; and SH: 103) meeting the inclusion criteria were included in the study. The baseline clinical data of patients are presented in Supplementary Table S1.

### Delineate the Mutation Landscape

Somatic mutation and clinical information were processed using R software. The “maftools” R package was further used to visualize the mutation oncoplot (Liu et al., 2021b). For each independent cohort, the mutation oncoplot displayed the genes with top 20 mutation frequency, which were defined as FMGs. The intersection genes of FMGs in the three cohorts were defined as the common FMGs.

### Assessment of Tumor Mutation Burden

TMB was defined as the total number of base substitutions, insertions, and deletions in the coding region per megabase (Liu et al., 2021c). Using the “tmb” function in the “maftool” R package, we calculated the TMB of each patient. All based substitutions and indels in the coding region of targeted genomes were retained. In contrast, synonymous mutations failing to contribute to amino acid change were discarded.

### Clinical Characteristics and Prognostic Evaluation

Univariate and multivariate Cox regression analyses were used for survival analysis of clinical characteristics of patients, including age, gender, hepatitis B virus (HBV), etc. Kaplan–Meier survival analysis was used to estimate the association between mutation phenotype and OS. Multiple boxplots were used to display differences in TMB among patients with the three phenotypes. In addition, to compare the clinical characteristics of patients with the three phenotypes in ICGC cohorts, we combined some clinical features to facilitate comparison. For example, I, IA, and IB stage (AJCC stages) were collectively referred to as I stage.

### Drug-Response Prediction

To explore the therapeutic response of different drugs, we downloaded the gene mutation and drug sensitivity information from the Genomics of Drug Sensitivity in Cancer (GDSC, https://www.cancerrxgene.org/). The sensitivity of different drugs was assessed by the half-maximal inhibitory (IC50), and the higher the IC50, the lower the sensitivity. Using our previous integrated pipeline (Liu et al., 2021c), we compare the drug sensitivity of different phenotypes. A summary is as follows: 1) Kolmogorov–Smirnov tests, a normality test algorithm, indicated that the imputed drug response (IC50) data were not normally distributed (*p* < 0.05). 2) Based on this result, Kruskal–Wallis and Wilcoxon rank-sum tests were utilized to calculate the *p*-values and the Benjamini–Hochberg (BH) method was used for multiple testing correction. 3) For each potential drug, if one phenotype was significantly lower than other phenotypes (Wilcoxon rank-sum and Kruskal–Wallis test, false discovery rate (FDR) < 0.05), the phenotype were defined as more sensitive to the drug. 4) The sensitivity of the three phenotypes was designated “Low sensitivity,” “Intermediate sensitivity,” and “High sensitivity” according to the magnitude of the median IC50 value.

### Statistical Analysis

All data processing, statistical analysis, and plotting were performed in R 4.0.5 software. The Wilcoxon rank-sum and Kruskal–Wallis tests were performed to compare the differences of two and multiple groups, respectively. Comparisons between categorical variables using Fisher’s exact test or chi-squared test were carried out. The Benjamin–Hochberg method was used to further calculate the FDR. For every analysis, statistical significance was considered at *p* < 0.05.

## Results

### Landscape of Somatic Mutations in ICC

**FIGURE 1:**
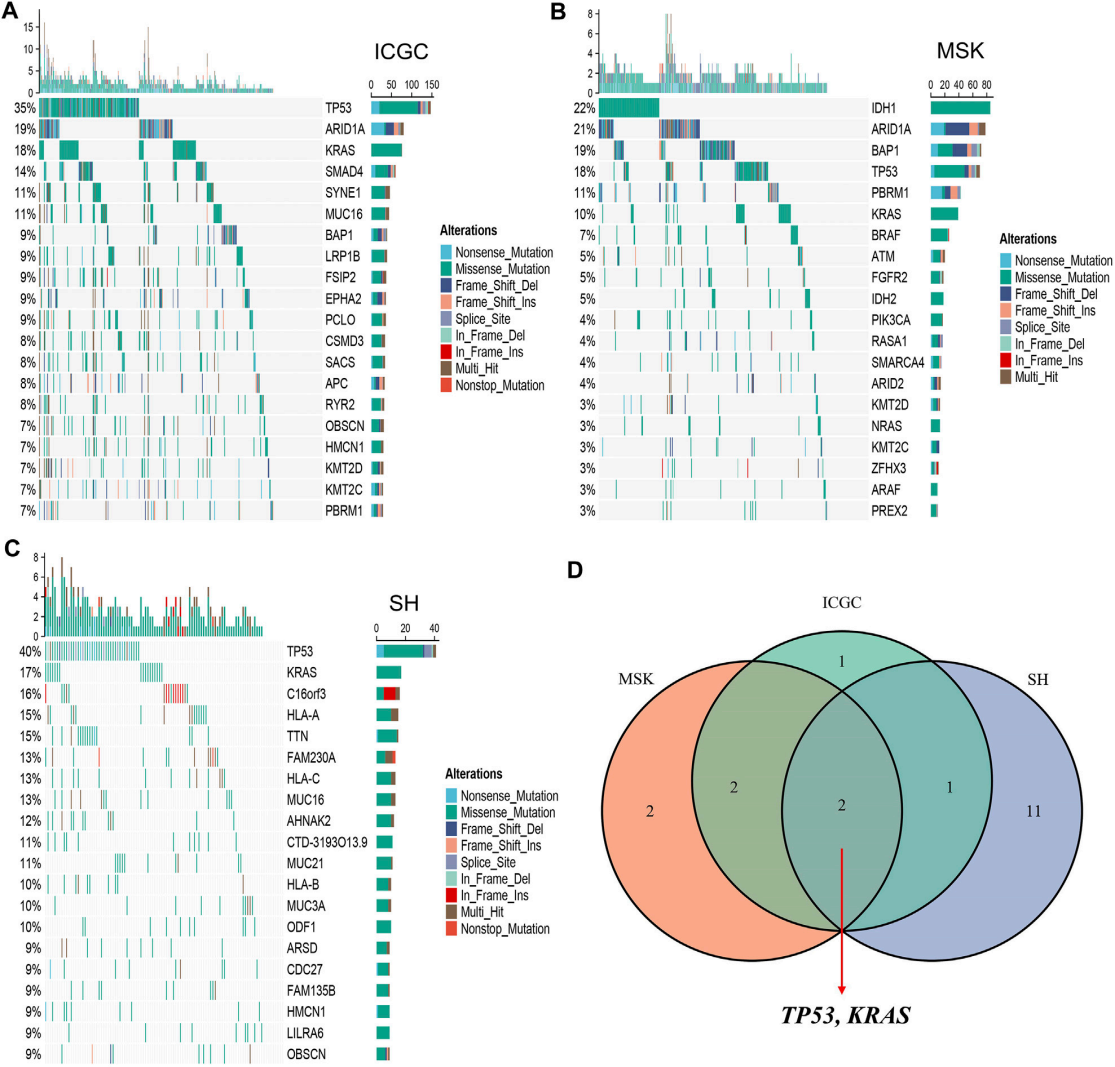
Landscapes of high-frequency mutated genes (FMGs) in intrahepatic cholangiocarcinoma (ICC). **(A–C)** Oncoplot depicts the FMGs of ICC in the ICGC **(A)**, MSK **(B)**, and Shanghai **(C)** cohorts. **(D)** Venn diagram of FMGs covered by the three large-scale cohorts.

The waterfall plot was utilized to describe the landscape of somatic mutations in ICC patients. We defined 20 FMGs in ICC samples from the ICGC cohort, which were TP53 (35%), ARID1A (19%), KRAS (18%), SMAD4 (14%), SYNE1 (11%), MUC16 (11%), BAP1 (9%), LRP1B (9%), FSIP2 (9%), and EPHA2 (9%) (Figure 1A). A total of 20 FMGs were also defined in ICC samples from the MSK cohort, including IDH1 (22%), ARID1A (21%), BAP1 (19%), TP53 (18%), PBRM1 (11%), KRAS (10%), BRAF (7%), ATM (5%), FGFR2 (5%), and IDH2 (5%) (Figure 1B). In addition, we also defined 20 FMGs in ICC samples from the Shanghai cohort, including TP53 (40%), KRAS (17%), C16orf3 (16%), HLA-A (15%), TTN (15%), FAM230A (13%), HLA-C (13%), MUC16 (13%), AHNAK2 (12%), and CTD-3193O13.9 (11%) (Figure 1C). Interestingly, three cohorts shared some common FMGs, including TP53 and KRAS (Figure 1D). Consequently, the subsequent analysis focused on TP53 and KRAS mutations.

### TP53 and KRAS Mutations Associated With TMB and Survival Prognosis

**FIGURE 2:**
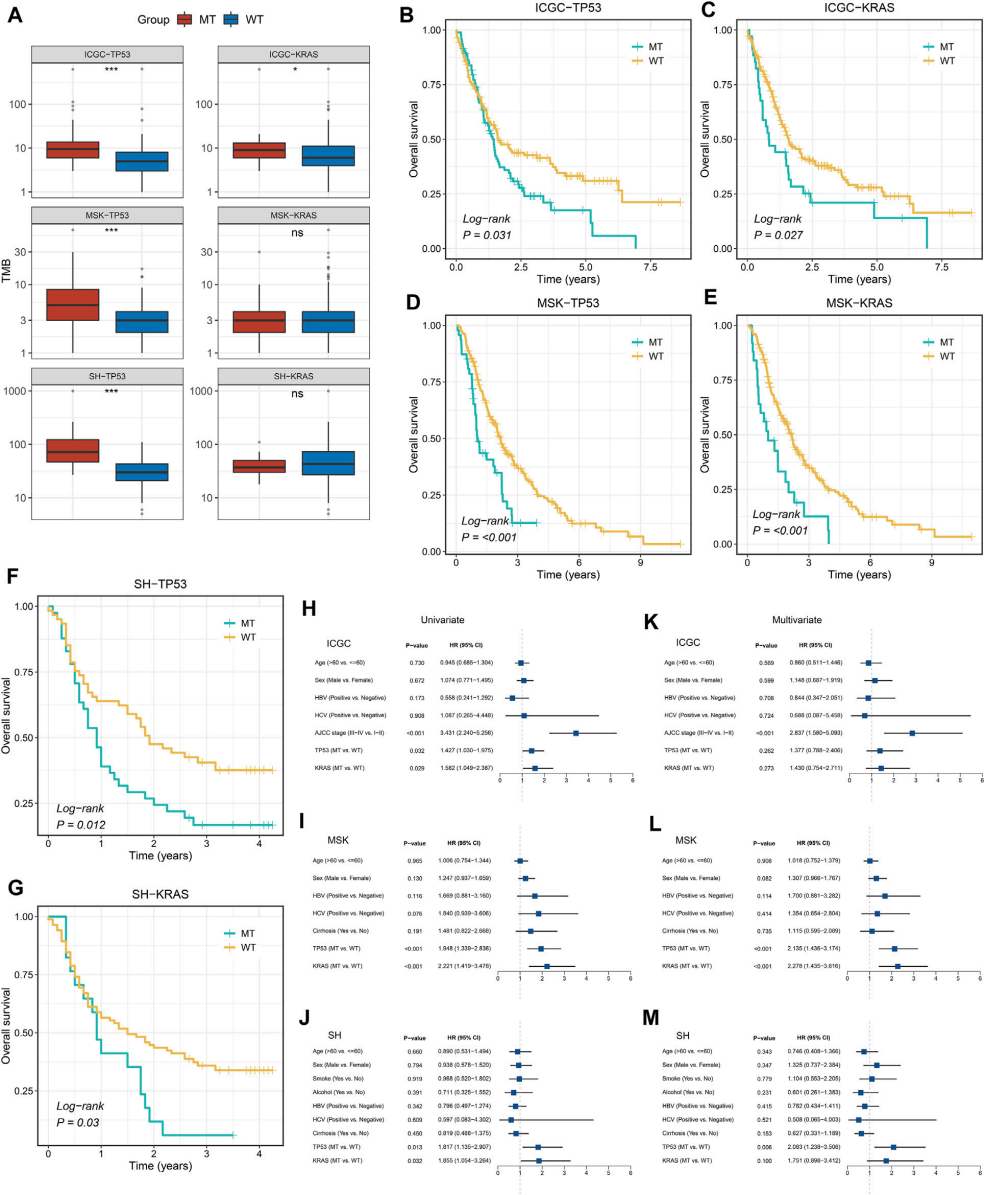
Gene mutations are associated with TMB and clinical prognosis. **(A)** TP53 and KRAS mutations are associated with a higher TMB. **(B–G)** Kaplan–Meier survival analysis of patients with TP53 or KRAS mutations in the three cohorts. **(H–M)** Univariate and multivariate Cox regression analysis. ns *p* > 0.05; *p < 0.05; **p < 0.01; ***p < 0.001.

Among the two common mutated genes, ICC patients with mutation in TP53 demonstrated significantly high TMB in the three cohorts (Figure 2A). Nevertheless, compared with patients without mutation in KRAS, patients with a mutation group only presented significantly high TMB in the ICGC cohort, which was not significantly different in the MSK and SH cohorts (Figure 2A). Subsequently, the Kaplan–Meier analysis was exploited to identify whether TP53 and KRAS mutations were associated with OS in ICC patients. As illustrated in Figures 2B–G, patients with TP53 and KRAS mutations presented a dismal prognosis. Univariate Cox regression analysis displayed that the hazard ratios (HRs) of TP53 and KRAS in the three cohorts (Figures 2H–J), respectively, were 1.427 (95% confidence interval [CI]: 1.030–1.975), 1.582 (95% CI: 1.049–2.387), 1.948 (95% CI: 1.339–2.836), 2.221 (95% CI: 1.419–3.478), 1.817 (95% CI: 1.135–2.907), and 1.855 (95% CI: 1.054–3.264) (all *p* < 0.05). Additionally, the multivariate analysis also indicated that TP53 and KRAS mutations remained statistically significant in the MSK cohort (all *p* < 0.05) (Figure 2L), and the HRs of TP53 and KRAS were 2.135 (95% CI: 1.436–3.174) and 2.278 (95%CI: 1.435–3.174). In the Shanghai cohort (Figure 2M), the HRs of TP53 and KRAS mutations were 2.083 (95% CI: 1.238–3.506, *p* < 0.05) and 1.751 (95% CI: 0.898–3.412, *P* = 0.10). However, TP53 and KRAS were also risk factors for prognosis in the ICGC cohorts, but the results were non-significant (Figure 2K).

### TP53/KRAS Mutation Phenotypes

**FIGURE 3:**
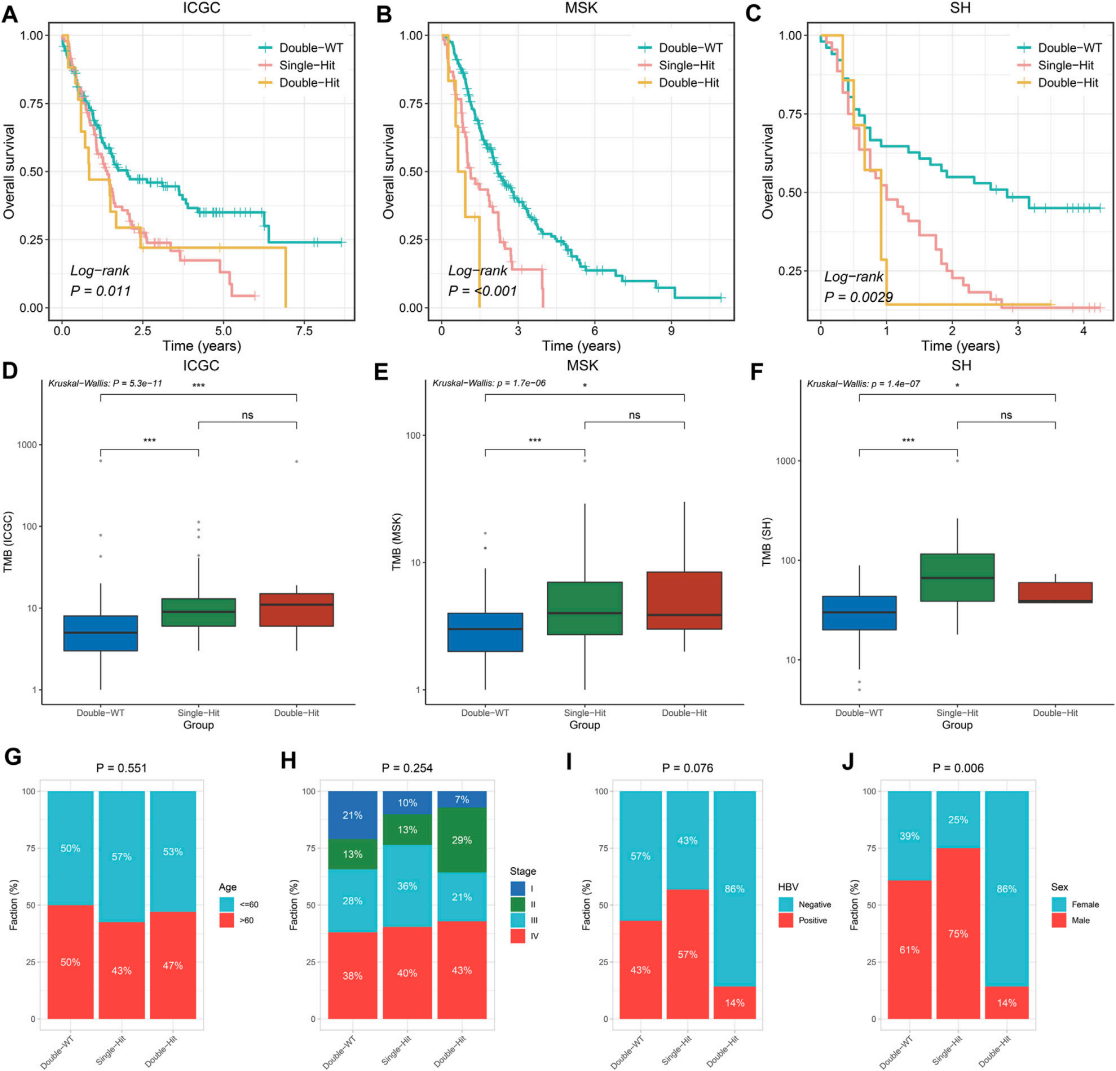
Difference of clinical characteristics and prognosis among three TP53/KRAS mutant phenotypes in the three cohorts. **(A–C)** Kaplan–Meier survival analysis of the three phenotypes. **(D–F)** Boxplot of TMB for patients with three phenotypes. **(G–J)** Composition percentage of Age **(G)**, AJCC stage **(H)**, HBV **(I)**, and Sex **(J)** among the three phenotypes. ns *p* > 0.05; *p < 0.05; **p < 0.01; ***p < 0.001.

**FIGURE 4:**
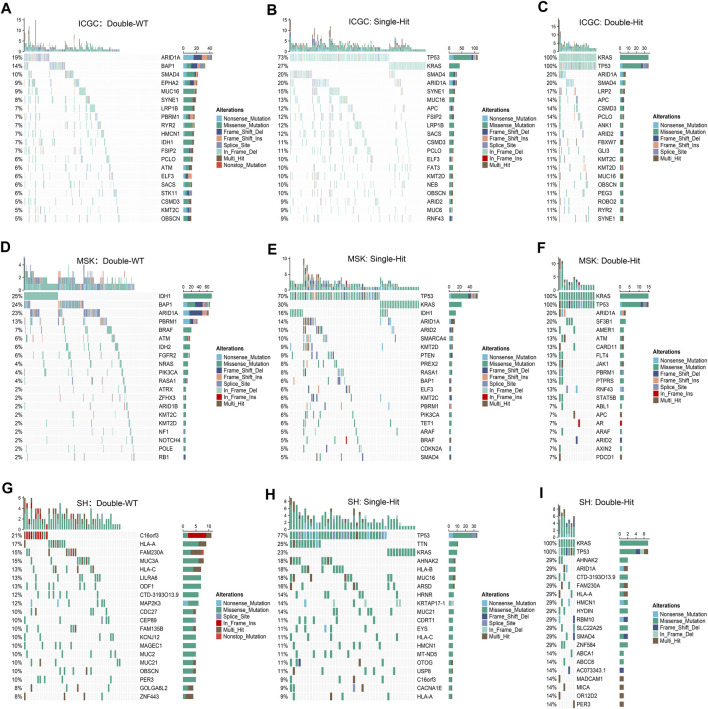
Mutation landscapes of “Double-WT,” “Single-Hit,” and “Double-Hit” phenotypes in the three cohorts. **(A–C)** In the ICGC cohorts, the mutation landscapes of “Double-WT” **(A)**, “Single-Hit” **(B)**, and “Double-Hit” **(C)** phenotypes. **(D–F)** Oncoplot depicts the FMGs for the three phenotypes in the MSK cohort. **(G–I)** Oncoplot depicts the FMGs for the three phenotypes in the SH cohort.

**FIGURE 5:**
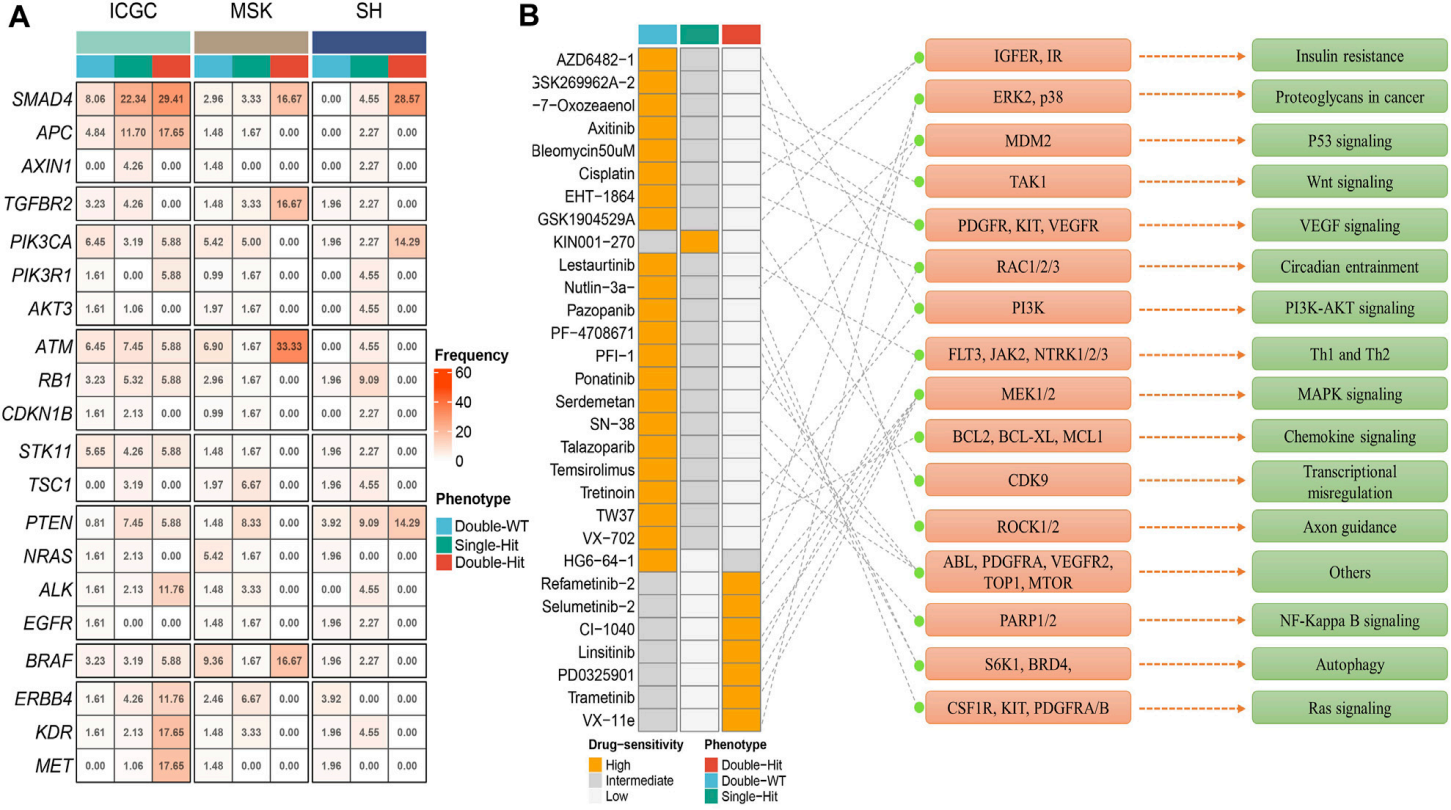
Molecular and pharmacological landscape of three mutant phenotypes. **(A)** Mutation rate of driver genes among three mutation phenotypes in the three cohorts. **(B)** 30 potential chemotherapy drugs with specific sensitivity to each phenotype were identified in total. The left panel represents the drug names and the level of sensitivity in each phenotype, the middle panel represents the drug-targeted molecules, and the right panel represents the drug-targeted pathways.

Prior studies have suggested that TP53 and KRAS mutation had a co-mutation phenomenon (Chen et al., 2021). Therefore, we suggested that the mutation status of TP53 and KRAS may be associated with clinical outcome and underlying biological characteristics of ICC patients. Based on the above considerations, patients with the double wild-type of TP53 and KRAS were labeled “Double-WT,” patients with one mutation (TP53 and KRAS) were labeled “Single-Hit,” and patients with the commutation of TP53 and KRAS were labeled “Double-Hit.” As showcased in Figures 3A–C, there were significant differences among the survival outcome among patients with three mutation subtypes in the three independent cohorts. Notably, patients’ OS becomes progressively shorter as TP53 and KRAS mutations accumulated. The “Double-Hit” phenotype patients had the shortest OS and the “Double-WT” phenotype patients had the longest OS, while the OS of “Single-Hit” phenotype patients was intermediate. Additionally, to further evaluate the prognostic values of the three phenotypes, the multivariable-adjusted analysis was utilized. As shown in Supplementary Figure S2, the “Double-WT” phenotype was an independent protective factor, while the “Single-Hit” and “Double-Hit” phenotypes were independent risk factors of prognosis. Subsequently, analysis of clinical characteristics in the ICGC cohort showed that there were no statistical differences in age, AJCC stage, and HBV status between the three subtypes (Figures 3G–I). In contrast, patients with the “Double-Hit” were more inclined to be female in the ICGC cohort (Figure 3J). Further comparison of TMB among the three phenotypes of patients revealed that the “Double-Hit” phenotype was a tendency toward higher, and significant differences were observed between the three phenotypes (Figures 3D–F). Waterfall plots of the three phenotypes suggest significant differences in the mutation landscapes of different phenotypes, and the “Double-Hit” phenotype had the lowest proportion (Figure 4A–I). Additionally, we calculated the frequencies of genes in the three phenotypes (Figure 5A), which were reported to be associated with the invasion and progression of cancer, such as SMAD4, APC, and ERBB4 (Zou et al., 2014; Lee et al., 2016). Noteworthily, patients with “Double-Hit” phenotype have higher mutation frequencies of SMAD4, APC, and AXIN1, which were numbers of the Wnt signaling pathway (Figure 5A). Previous study has reported that the Wnt signaling pathway contributed to the progression of cholangiocarcinoma by activating the downstream target genes (Zhang et al., 2020). BRAF mutation has been identified as a risk factor of cholangiocarcinoma (Tannapfel et al., 2003) and was most common in “Double-Hit” phenotype (Figure 5A).

### Assessment of Chemotherapy Sensitivity

Based on the mutation and drug sensitivity information obtained from the GDSC database, the responses of ICC patients with different phenotypes to 266 chemotherapeutic agents were compared, which contributed to exploring drugs with specific sensitivity to each phenotype. As illustrated in Figure 5B, we identified a total of 30 sensitive drugs for ICC patients, of which 22 drugs were sensitive to “Double-WT” (such as Axitinib, Cisplatin, Pazopanib, Lestaurtinib, and PFI-1 et al.), 7 drugs were sensitive to “Double-Hit” (such as Refametinib-2, Linsitinib, Trametinib, and VX-11e et al.), and only one drug was sensitive to “Single-Hit” (KIN001-270). Interestingly, the targets of sensitive drugs for “Double-Hit” phenotype patients mainly focused on the MAPK signaling pathway. Likewise, p53 signaling, VEGF signaling, and PI3K-AKT signaling were the targets of sensitive drugs for “Double-WT” patients. The drug sensitivity and target information may provide opportunities for targeted therapy in ICC patients with different phenotypes. Our study created conditions for chemotherapy for three mutation phenotypes.

## Discussion

In the current era of precision medicine, decoding the genetic information of tumors from the genetic levels is increasingly important for the treatment of ICC patients. In the present study, we comprehensively analyzed 899 clinical and genomics mutation data from ICGC, MSK, and Shanghai cohorts. TP53 and KRAS were common FMGs in ICC, and its mutation was associated with higher TMB and worse prognosis. Given the co-mutation phenomenon of TP53 and KRAS, three mutation phenotypes (“Double-WT,” “Single-Hit,” and “Double-Hit”) were identified in ICC patients. With the cumulative mutation number in the three phenotypes, the prognosis of patients showed a tendency of dismalness. Noteworthily, we unearthed multiple potentially sensitive chemotherapeutic drugs of every phenotype, which provided a resource for precise chemotherapy of ICC patients in the clinic. In summary, our works presented a novel mutation classification and elucidated the importance of FMGs in guiding the treatment of ICC patients.

KRAS and TP53 mutations were known as major driver oncogenes in a variety of cancers, including pancreatic ductal carcinoma, non–small-cell lung cancer, and high-grade serous carcinoma (Bange et al., 2019; Sauriol et al., 2020; Tsutaho et al., 2020). Nevertheless, the clinical significance and molecular mechanism of this co-mutation phenomenon in ICC have not been elaborated. In our research, we found that TP53 and KRAS were the FMGs in cohorts from different countries. This suggests that the phenomenon of TP53 and KRAS high-frequency mutations is not affected by race and sequencing platforms, which is important for the research of ICC. A previous study reported that mutation of TP53 would cause the download of p53, which is a tumor suppressor (Shi and Jiang, 2021). Dysfunction of p53 affects the T cell activation, which plays a key role in tumor immune escape. Similarly, KRAS mutation reduces tumor immunogenicity by inhibiting tumor neoantigen accumulation, thereby promoting tumor progression (Frost et al., 2021; Tran et al., 2021). Unsurprisingly, univariate and multivariate analysis displayed that TP53 and KRAS mutations were risk factors in multiple ICC cohorts. The prognosis of patients with the three phenotypes of “Double-WT,” “Single-Hit,” and “Double-Hit” was significantly indifferent, with “Double-Hit” having the worst prognosis and “Double-WT” having the best prognosis, which suggests an accumulative effect of the two mutations.

In addition, we found that we found that TMB tended to increase with the accumulation of TP53 and KRAS mutations in the ICGC and MSK cohort. However, due to the small number of patients in the “Double-Hit” group, the increase in TMB was not significant (ICGC cohort and MSK cohort) or even decreased (SH cohort) in the “Double-Hit” group compared with the “Single-Hit” and “Double-WT” groups. TMB quantifies the mutations found in the tumor and is correlated with quantity of neoantigens (Büttner et al., 2019; Grosser et al., 2019). Evidence indicated that patients with higher TMB also carry higher neoantigen loads (Büttner et al., 2019). This suggested that patients with “Double-Hit” (who tend to experience increase in TMB in the ICGC and MSK cohorts) are a potentially beneficial population for immunotherapy. In this study, we also found potentially sensitive chemotherapeutic agents for patients with different phenotypes. Patients with “Double-WT” phenotype were more sensitive to Axitinib, Cisplatin, and PFI-1. Likewise, patients with “Double-Hit” and “Single-Hit” phenotype also benefited from specific drugs, such as Trametinib and KIN001-270. Combining the benefits of immunotherapy and chemotherapy, our work provides guidance for the clinical management and individualized treatment of ICC patients with different phenotypes. However, this study has shortcomings, which are as follows: 1) further randomized clinical trials are necessary to validate the study findings and 2) some patients lacked clinical features, such as AJCC, tumor size, and lymph node metastasis. Although our results were derived from bioinformatics analysis rather than clinical experiments, we believe that comprehensive analysis based on the multicenter and larger sample can compensate for the shortcoming.

## Conclusion

In conclusion, we defined a novel classification based on the common FMGs (TP53 and KRAS) in three large-scale cohorts. Patients with the three phenotypes showed significant differences in mutation landscape, prognosis, and pharmacological sensitivity, which may provide new insights for individualized treatment and management of ICC patients.

## Data Availability

The original contributions presented in the study are included in the article/Supplementary Material; further inquiries can be directed to the corresponding authors.
