# Supplementary material for: TP53 /KRAS Co-Mutations Create Divergent Prognosis Signatures in Intrahepatic Cholangiocarcinoma
Source: Front Genet. 2022 Mar 25;13:844800. doi: 10.3389/fgene.2022.844800 (PMC8990229; doi:10.3389/fgene.2022.844800)
Supplement: Supplementary file 1 [file DataSheet1.docx]

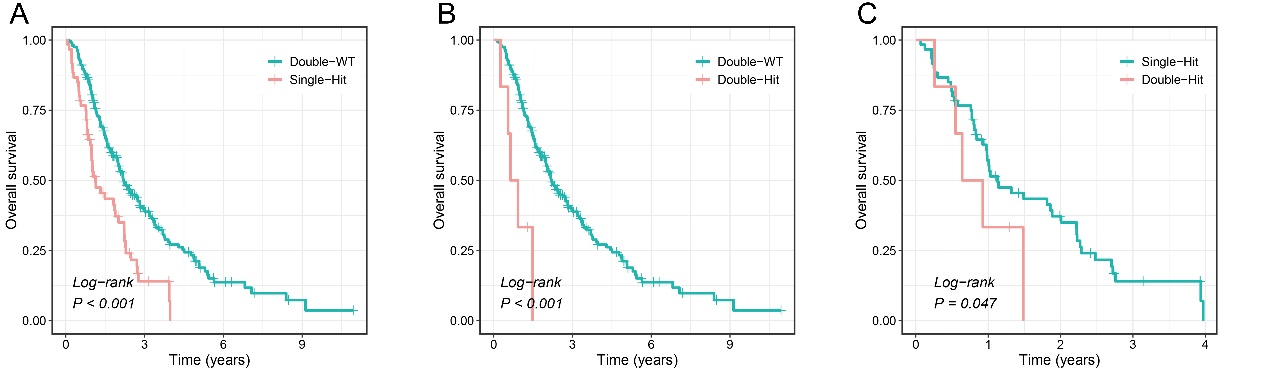


**Figure S1: Kaplan-Meier survival analysis of patients with three phenotypes in the MSK cohort. A** Kaplan-Meier curve of ‘Double-WT’ and ‘Single-Hit’**. B** Kaplan-Meier curve of ‘Double-WT’ and ‘Double-Hit’**. C** Kaplan-Meier curve of ‘Single-Hit’ and ‘Double-Hit’**.**

**

**

**Figure S2: The multivariate Cox regression analysis of the three phenotypes in the three cohorts.** (**A**) ICGC cohort: Double-WT phenotype; (**B**) ICGC cohort: Single-Hit phenotype; (**C**) ICGC cohort: Double-Hit phenotype; (**D**) MSK cohort: Double-WT phenotype; (**E**) MSK cohort: Single-Hit phenotype; (**F**) MSK cohort: Double-Hit phenotype; (**G**) SH cohort: Double-WT phenotype; (**H**) SH cohort: Single-Hit phenotype; (**I**) SH cohort: Double-Hit phenotype.
